# Supplementary material for: Naphthalimide-Piperazine Derivatives as Multifunctional “On” and “Off” Fluorescent Switches for pH, Hg2+ and Cu2+ Ions
Source: Molecules. 2023 Jan 28;28(3):1275. doi: 10.3390/molecules28031275 (PMC9918953; doi:10.3390/molecules28031275)
Supplement: Supplementary file 1 [file molecules-28-01275-s001.zip › molecules-2116686-supplementary.pdf]

Supplementary Materials

Naphthalimide-piperazine derivatives as multifunctional "on" and "off" fluorescent switches for pH, Hg^{2+} and Cu^{2+} ions

Kristina Pršir [1], Mislav Matić [1], Marlena Grbić [1], Gerhard J. Mohr [2], Svjetlana Krištafor [1] and Ivana Murković Steinberg [1,*]

[1] Department of General and Inorganic Chemistry, Faculty of Chemical Engineering and Technology, University of Zagreb, Marulićev trg 19, 10000 Zagreb, Croatia; kbobanov@fkit.hr (K.P.); mmatic@fkit.hr (M.M); marlena.grbic@iqvia.com (M.G.); prekupec@fkit.hr (S.K.)

[2] Joanneum Research Forschungsgesellschaft mbH – Materials, Franz-Pichler-Straße 30, Weiz A-8160, Austria; gerhard.mohr@joanneum.at (G.J.M.)

* Correspondence: imurkov@fkit.hr (I.M.S.)

Figure S1. Absorbance spectra of: a) **NI-1** and c) **NI-2** in a buffer/methanol mixture (2:1) at different pH; plot of absorbance ratio at 388 nm and 410 nm vs. pH for: b) **NI-1** and d) **NI-2**.

Figure S2. a) Fluorescence spectra of **NI-2** in buffer methanol mixture (2:1) at different pH; b) fluorescence intensity at 525 nm vs. pH.

Figure S3. NI-1 in the presence of metal ions (from left: blank, Zn^{2+}, Fe^{2+}, Fe^{3+}, Cu^{2+}, Al^{3+}, Hg^{2+}, Pb^{2+}, Co^{2+}, Ni^{2+}, Ag^{+}, Mn^{2+}, Ba^{2+}, Cd^{2+}, Mg^{2+}, Ca^{2+}) c (metal ion) = 3.0×10^{-5} M.

Figure S4. UV-Vis spectra of **NI-1** solution upon the addition of 20 equiv. of a) Hg(II) and b) Cu(II) ions.

Figure S5. Changes in the emission spectra of **NI-1** upon gradual addition of Cu^{2+} and EDTA.

Figure S6. Changes in the emission spectra of **NI-1** upon gradual addition of Hg^{2+} and EDTA.

Figure S7. Benesi-Hildebrand's plot in linear range for **NI-1** binding with a) Cu^{2+} b) Hg^{2+}.

Figure S8. Dependence of fluorescence intensity of **NI-1** (5.00×10^{-7} M) in MeOH / buffer pH 5.5 (v / v, 1/2) at 523 nm on the ion concentration in the linear range of dependence for a) Cu^{2+} b) Hg^{2+} ion.

Figure S9. Stern-Volmer plot of the ratio of the fluorescence intensities (F_0/F) of **NI-1** against the increasing concentration of a) Cu^{2+} and b) Hg^{2+} ions in linear range.

Figure S10. UV-Vis spectra of **NI-2** upon the addition of Hg(II) ions.

Figure S11. Job's plot fluorescence intensity at 525 nm on the molar content of **NI-2** in presence of Hg^{2+} ions at a total concentration of 5.00×10^{-6} M. The extrapolated directions of intensity dependence on the molar fraction of **NI-2** are presented.

Figure S12. Benesi-Hildebrand's plot in linear range for **NI-2** binding with Hg^{2+}.

Figure S13. Dependence of fluorescence intensity of **NI-2** (6.53×10^{-6} M) in MeOH / buffer 5.5 (v/v, 1/2) at 525 nm on the ion concentration in the linear range of dependence for Hg^{2+} ion.

Figure S14. Reversibility test for **NI-2** and Hg(II) binding.

a)

b)

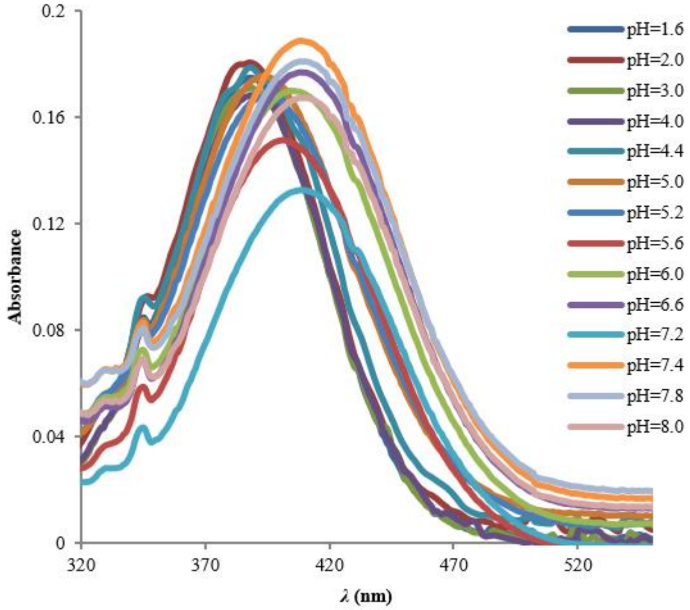
0.2
0.16
0.12
0.08
0.04
0
Absorbance
320
370
420
470
520
λ (nm)
pH=1.6
pH=2.0
pH=3.0
pH=4.0
pH=4.4
pH=5.0
pH=5.2
pH=5.6
pH=6.0
pH=6.6
pH=7.2
pH=7.4
pH=7.8
pH=8.0

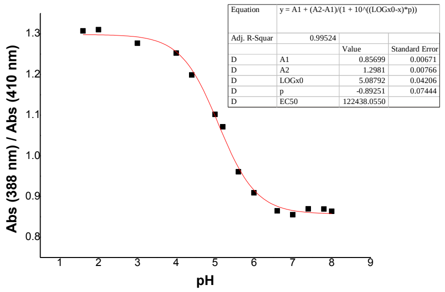
Equation | y = A1 + (A2-A1)/(1 + 10^((LOGx0-x)*p))
Adj. R-Squar | 0.99524
Value | Standard Error
D | A1 | 0.85699 | 0.00671
D | A2 | 1.2981 | 0.00766
D | LOGx0 | 5.08792 | 0.04206
D | p | -0.89251 | 0.07444
D | EC50 | 122438.0550
Abs (388 nm) / Abs (410 nm)
1.3
1.2
1.1
1.0
0.9
0.8
1 2 3 4 5 6 7 8 9
pH

c)

d)

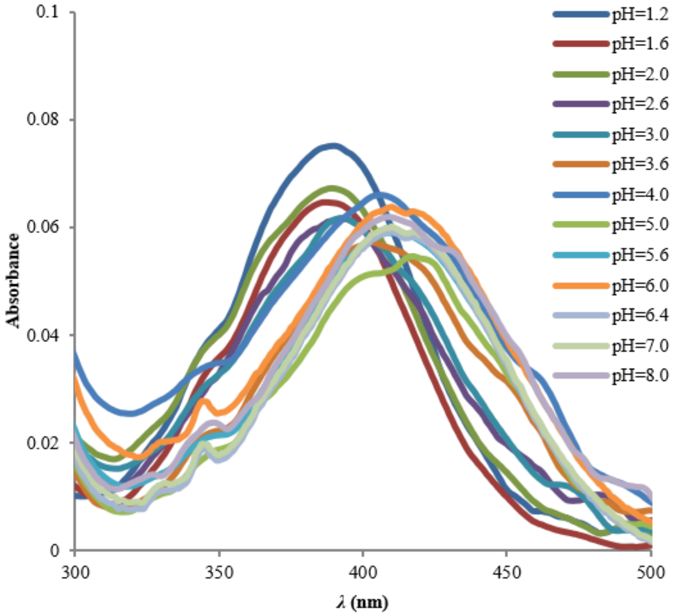
0.1
0.08
0.06
0.04
0.02
0
Absorbance
300
350
400
450
500
λ (nm)
pH=1.2
pH=1.6
pH=2.0
pH=2.6
pH=3.0
pH=3.6
pH=4.0
pH=5.0
pH=5.6
pH=6.0
pH=6.4
pH=7.0
pH=8.0

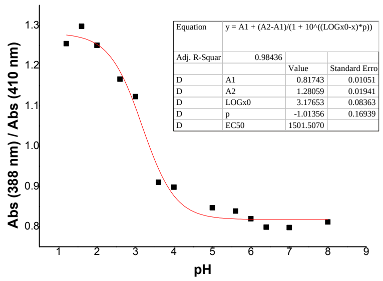
Equation | y = A1 + (A2-A1)/(1 + 10^((LOGx0-x)*p))
Adj. R-Squar | 0.98436
Value | Standard Erro
D | A1 | 0.81743 | 0.01051
D | A2 | 1.28059 | 0.01941
D | LOGx0 | 3.17653 | 0.08363
D | p | -1.01356 | 0.16939
D | EC50 | 1501.5070
Abs (388 nm) / Abs (410 nm)
1.3
1.2
1.1
1.0
0.9
0.8
1 2 3 4 5 6 7 8 9
pH

Figure S1. Absorbance spectra of: a) **NI-1** and c) **NI-2** in a buffer/methanol mixture (2:1) at different pH; plot of absorbance ratio at 388 nm and 410 nm vs. pH for: b) **NI-1** and d) **NI-2**. The spectra of compounds **NI-1** and **NI-2** were recorded in MeOH/buffer (1:2) at compound concentrations 6.56×10^{-5} M and 6.54×10^{-5} M, respectively.

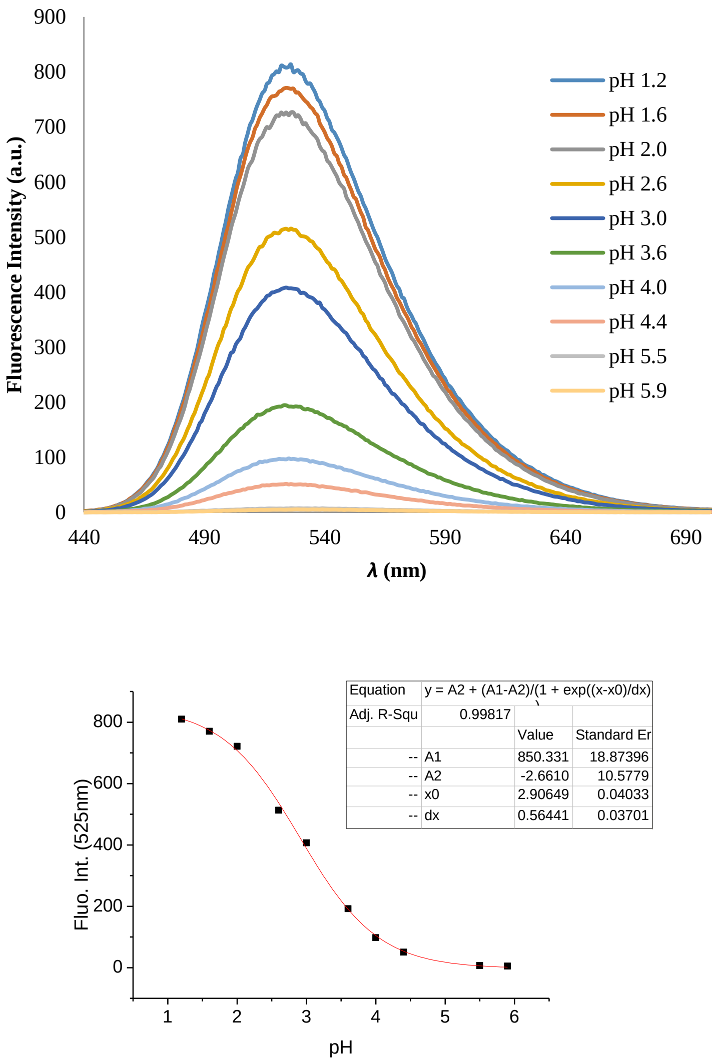

Figure S2. a) Fluorescence spectra of **NI-2** in buffer methanol mixture (2:1) at different pH; b) fluorescence intensity at 525 nm vs. pH.

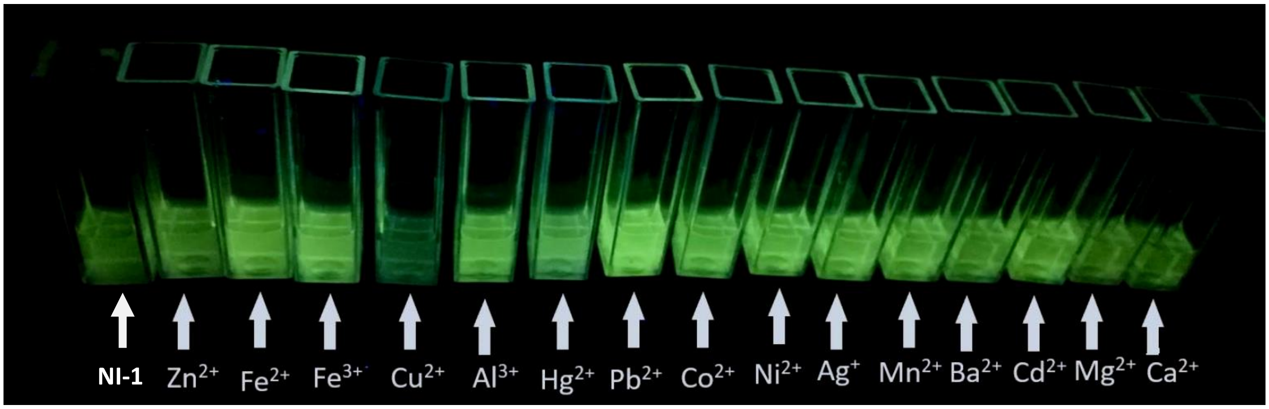

Figure S3. NI-1 in the presence of metal ions (from left: blank, Zn^{2+}, Fe^{2+}, Fe^{3+}, Cu^{2+}, Al^{3+}, Hg^{2+}, Pb^{2+}, Co^{2+}, Ni^{2+}, Ag^{+}, Mn^{2+}, Ba^{2+}, Cd^{2+}, Mg^{2+}, Ca^{2+}) c (metal ion) = 3.0×10^{-5} M.

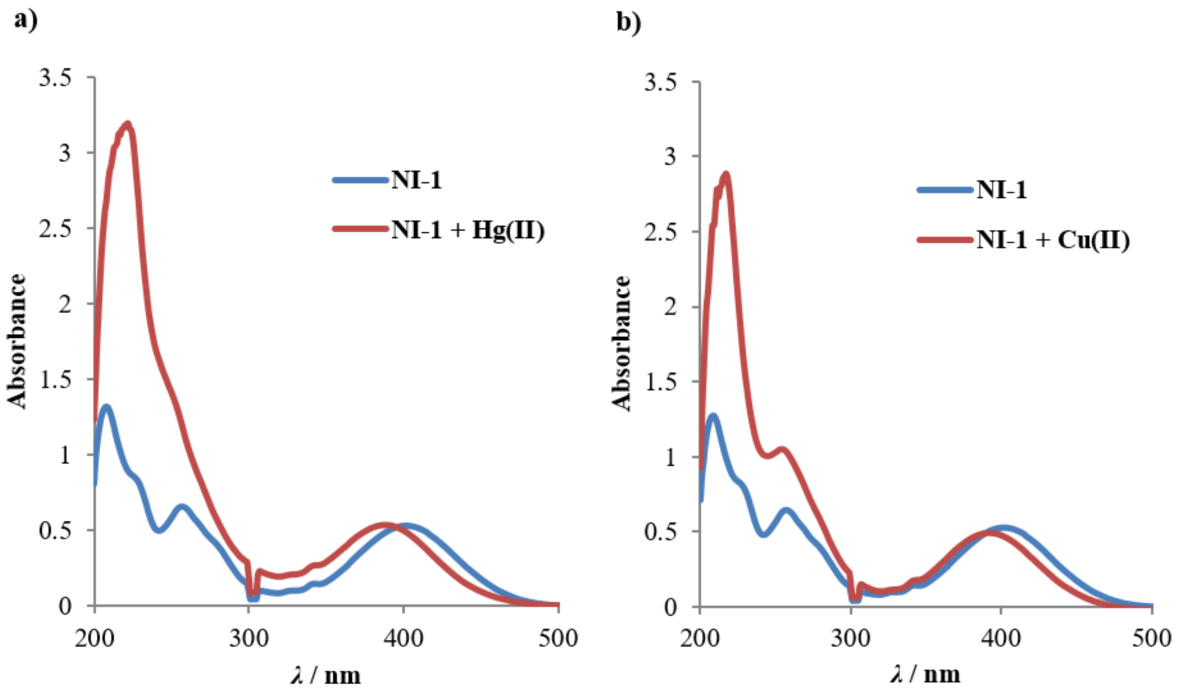

Figure S4. UV-Vis spectra of **NI-1** solution upon the addition of 20 equiv. of a) Hg(II) and b) Cu(II) ions.

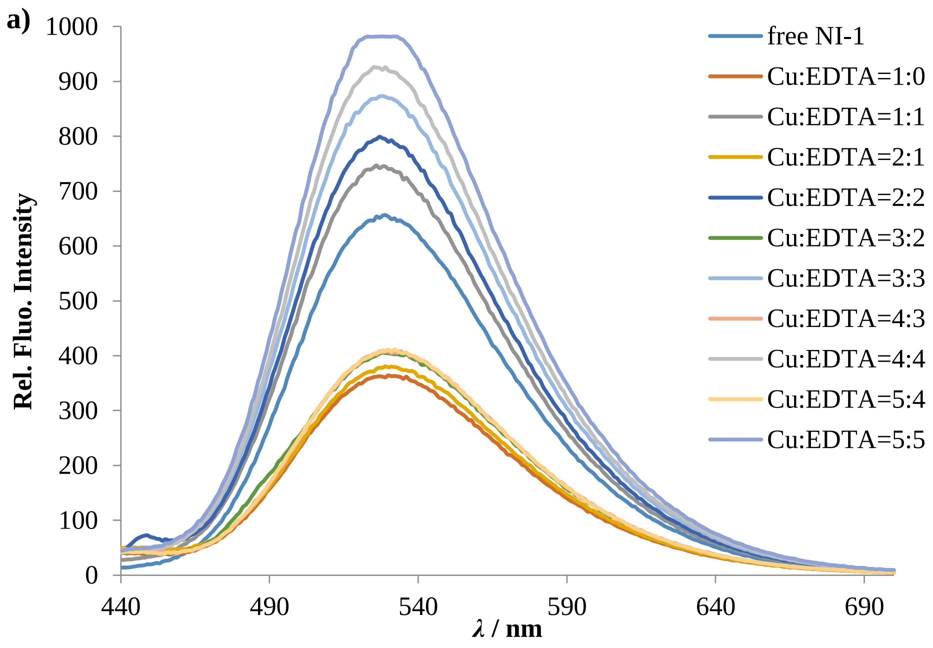

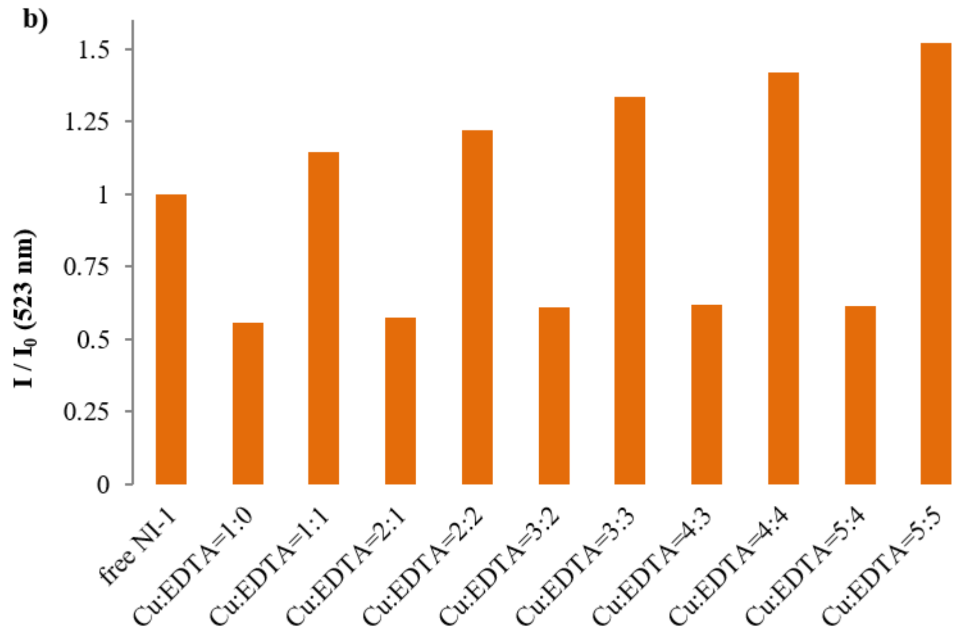

Figure S5. Changes in the emission spectra of **NI-1** upon gradual addition of Cu^{2+} and EDTA.

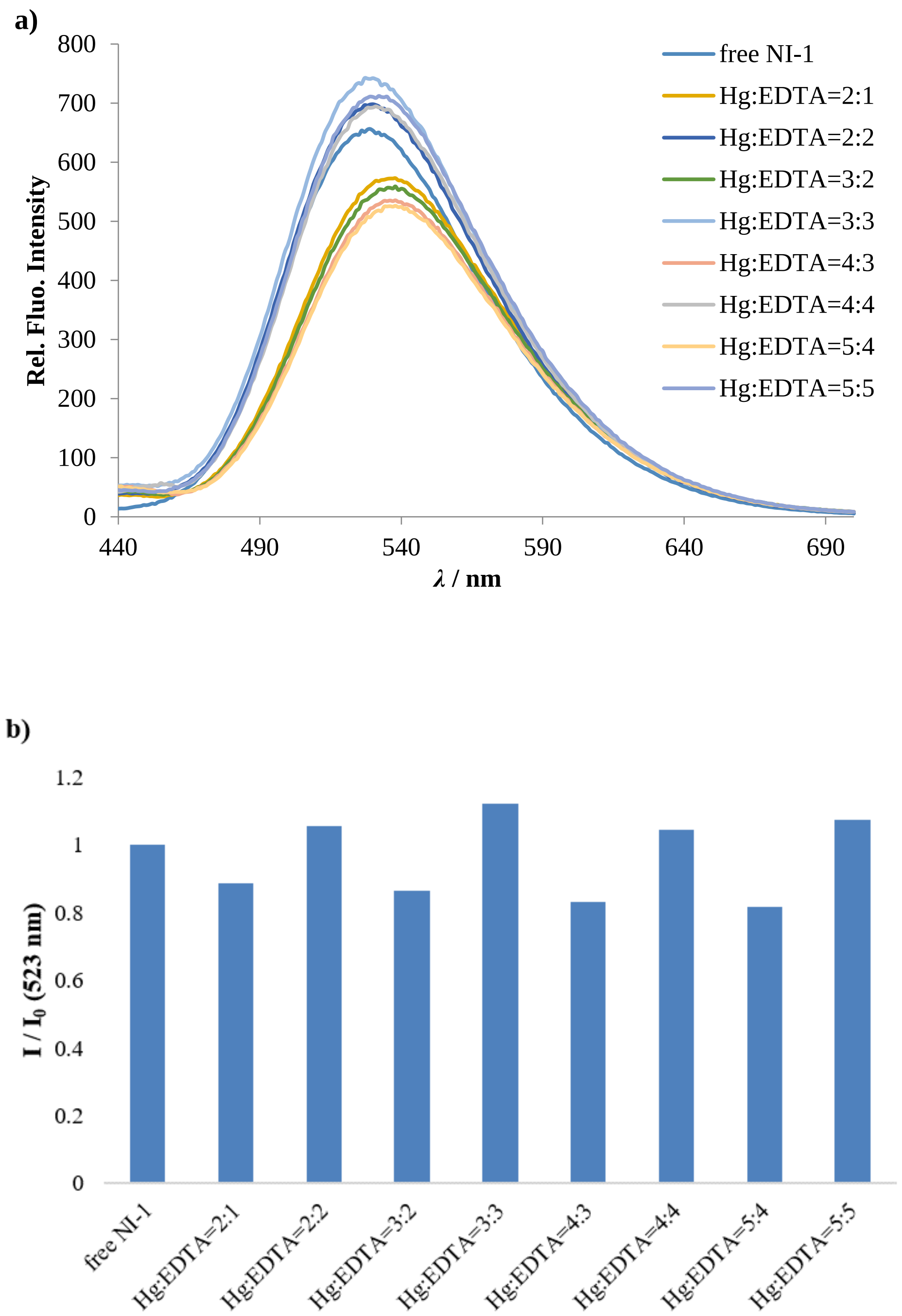

Figure S6. Changes in the emission spectra of **NI-1** upon gradual addition of Hg^{2+} and EDTA.

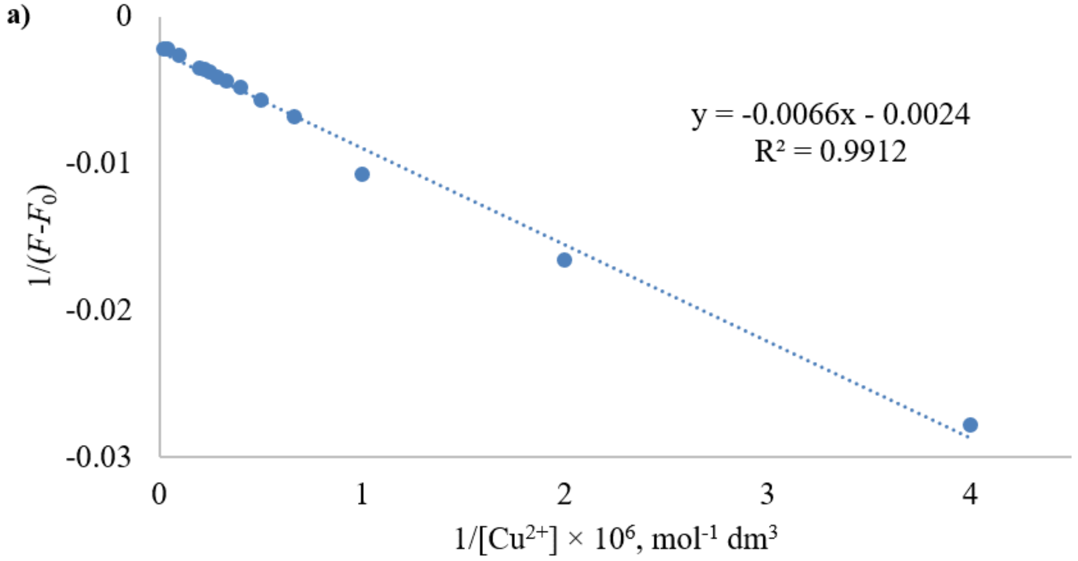

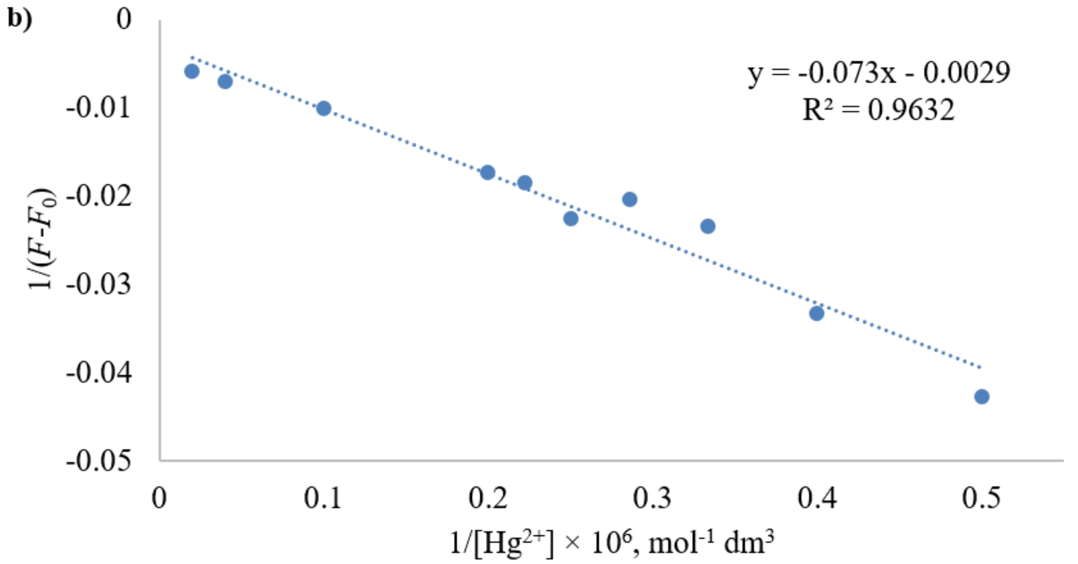

Figure S7. Benesi-Hildebrand's plot in linear range for **NI-1** binding with a) Cu^{2+} b) Hg^{2+}.

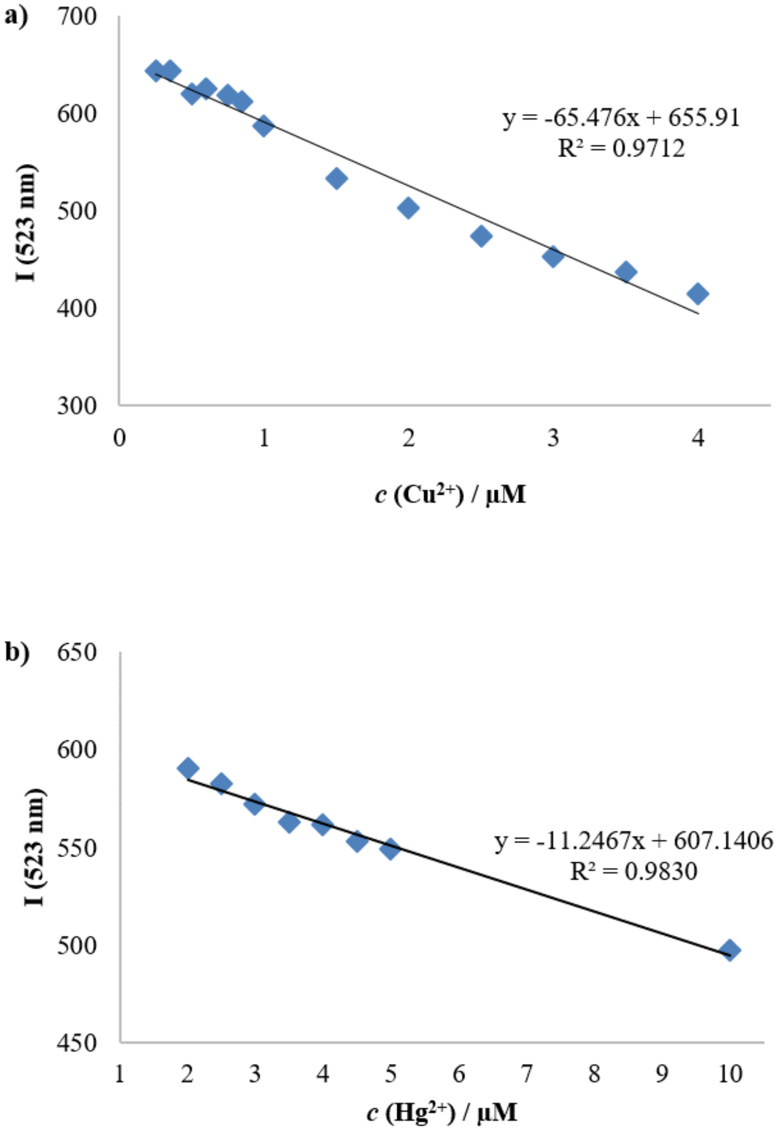

Figure S8. Dependence of fluorescence intensity of **NI-1** (5.00×10^{-7} M) in MeOH / buffer pH 5.5 (v / v, 1/2) at 523 nm on the ion concentration in the linear range of dependence for a) Cu^{2+} b) Hg^{2+} ion.

Stern-Volmer's equation for **NI-1** was derived using:

$$\frac{F_0}{F} = 1 + K_{sv}[Q]$$

where F_0 - initial fluorescence intensity, F – fluorescence intensity in the presence of the analyte, $[Q]$ – concentration of fluorescence quencher (Cu^{2+} or Hg^{2+} ion), K_{sv} – Stern-Volmer constant.

The Stern-Volmer constant is equal to the slope in the linear range of the fluorescence intensity ratio of **NI-1** without metal ions and fluorescence intensity in the presence of ions at 523 nm as shown in Fig. S10. For Cu^{2+} ion the Stern-Volmer constant is 152570 M^{-1}, and for Hg^{2+} ion it is 23909 M^{-1}.

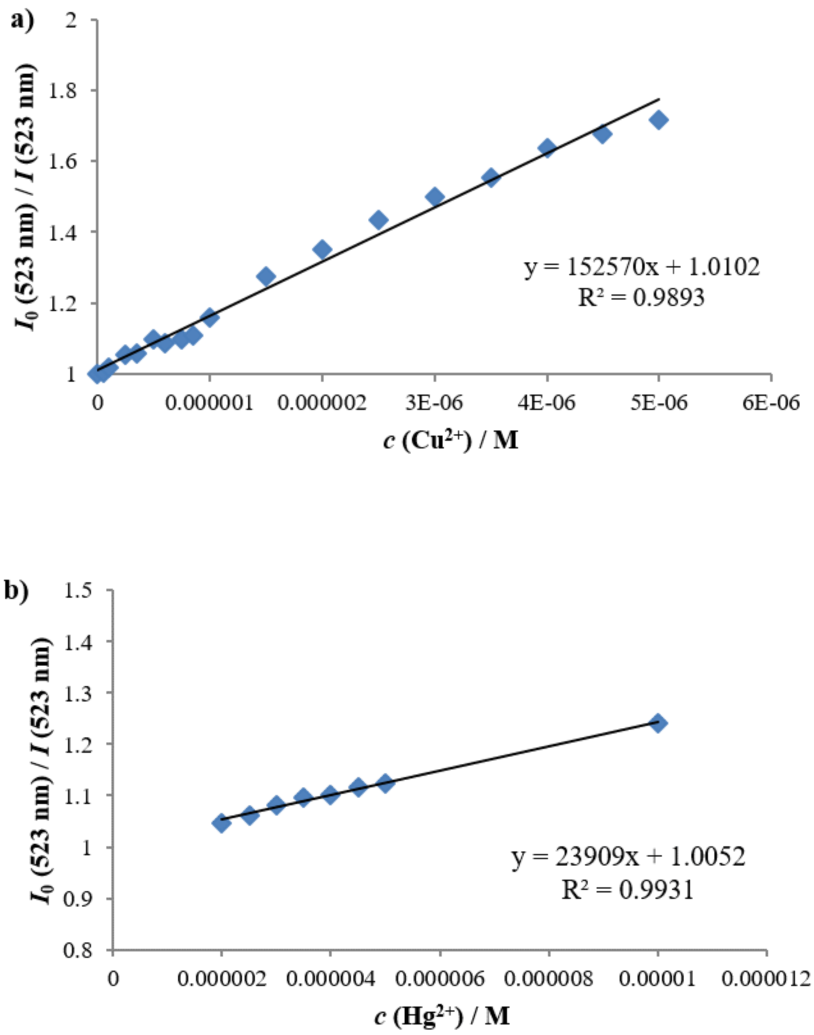

Figure S9. Stern-Volmer plot of the ratio of the fluorescence intensities (F_0/F) of **NI-1** against the increasing concentration of a) Cu^{2+} and b) Hg^{2+} ions in linear range.

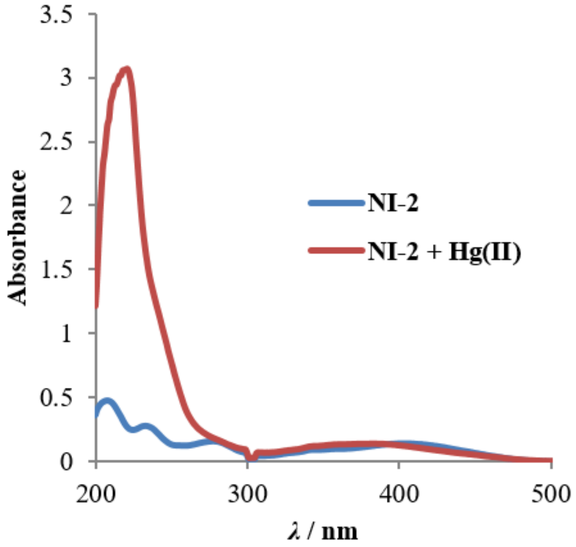

Figure S10. UV-Vis spectra of **NI-2** upon the addition of Hg(II) ions.

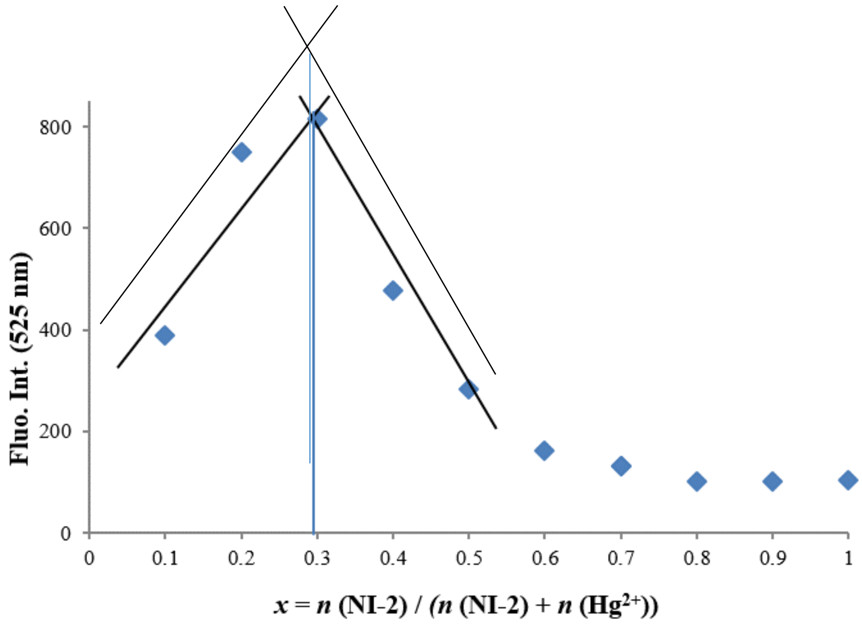

Figure S11. Job's plot fluorescence intensity at 525 nm on the molar content of **NI-2** in presence of Hg^{2+} ions at a total concentration of 5.00×10^{-6} M. The extrapolated directions of intensity dependence on the molar fraction of **NI-2** are presented.

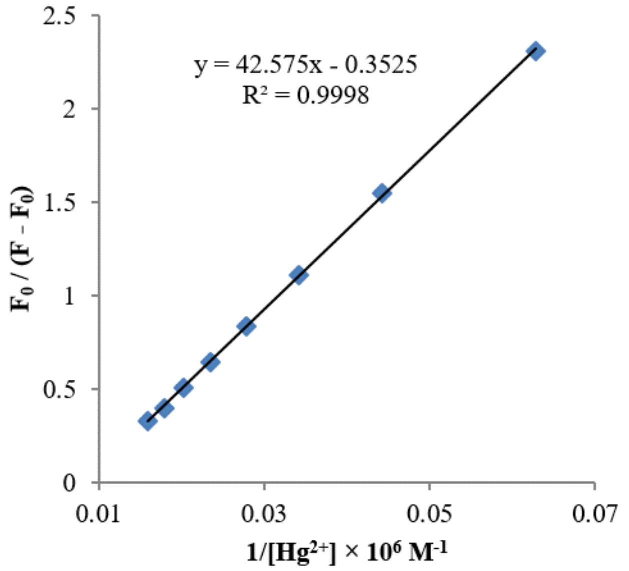

Figure S12. Benesi-Hildebrand's plot in linear range for **NI-2** binding with Hg^{2+}.

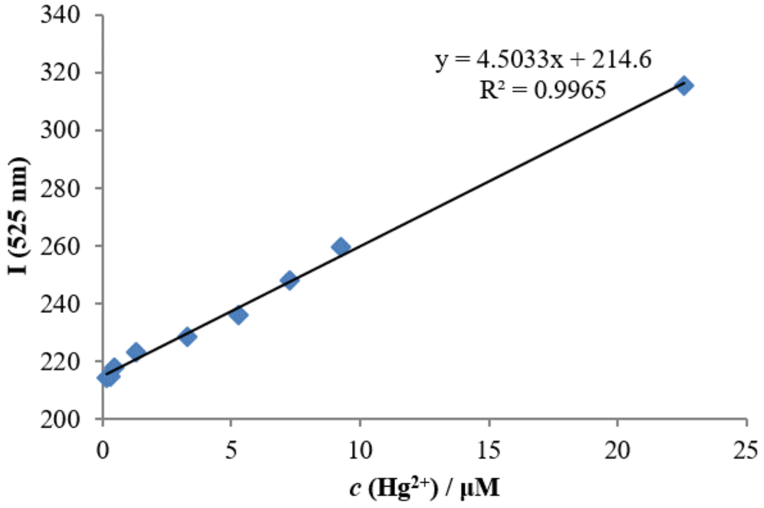

Figure S13. Dependence of fluorescence intensity of **NI-2** (6.53×10^{-6} M) in MeOH / buffer 5.5 (v/v, 1/2) at 525 nm on the ion concentration in the linear range of dependence for Hg^{2+} ion.

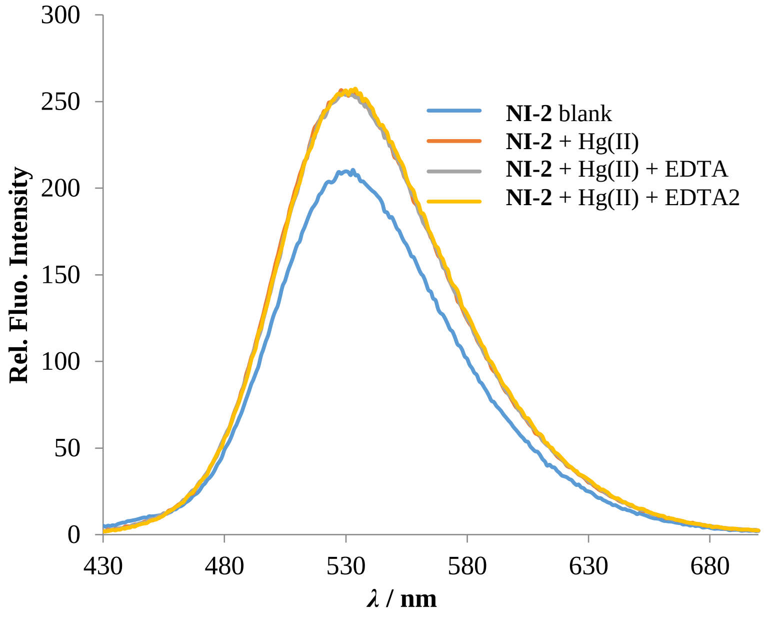

Figure S14. Reversibility test for **NI-2** and Hg(II) binding.
